# The temporal association between adverse drug reactions and antirheumatic drugs utilisation in Western Australia: a retrospective study from real-world data (1995–2015)

**DOI:** 10.1007/s00296-024-05588-3

**Published:** 2024-04-14

**Authors:** Khalid B. Almutairi, Charles A. Inderjeeth, David B. Preen, Helen I. Keen, Johannes C. Nossent

**Affiliations:** 1 School of Medicine, The University of Western Australia, Perth, Australia; 2 King Fahd Specialist Hospital, Burydah, Saudi Arabia; 3 Sir Charles Gairdner and Osborne Park Health Care Group, Perth, Australia; 4 School of Population and Global Health, University of Western Australia, Perth, WA Australia; 5 Fiona Stanley Hospital, Murdoch, WA Australia

**Keywords:** Pharmacovigilance, Adverse drug reactions, Western Australia database of adverse event notifications, Disease-modifying antirheumatic drugs, United States Food and Drug Administration Adverse Event Reporting System

## Abstract

**Background/Objectives:**

Adverse drug reactions (ADRs) can result in morbidity, mortality, and higher healthcare costs. Given the limited information available on ADRs associated with antirheumatic medications, this study aims to analyse and compare ADR reporting for these drugs in the pharmacovigilance datasets of Western Australia (WA) and the United States (US).

**Methods:**

Therapeutic Goods Administration provided WA pharmacovigilance data of selected antirheumatic drugs to from 1995 to 2015. The proportional reporting ratio (PRR) for WA case reports was compared to corresponding USA pharmacovigilance data by assessing the disproportionality of each ADR. clinically significant or true ADRs were determined using the Evans 2001 criteria (*n* > 2, chi-square > 4, PRR > 2).

**Results:**

A total of 232 reports were found in WA, mostly on sixty-nine women aged 45 to 69. Methotrexate, leflunomide, azathioprine, sulfasalazine, and infliximab had the highest reported ADRs, related to gastrointestinal disorders. Patients who used biological agents in WA had 2.7 times the likelihood of reporting true ADRs compared to conventional antirheumatic drugs. The ADR rates in the two datasets were comparable over the study period.

**Conclusions:**

The PRR values of ADRs were consistent between WA and US databases. Methotrexate and infliximab use were commonly associated with ADR reports in WA females, with incidence rates comparable to the US; while patients using biological agents were more likely to report true ADRs than those on conventional antirheumatic drugs in WA.

**Supplementary Information:**

The online version contains supplementary material available at 10.1007/s00296-024-05588-3.

## Introduction

Adverse drug reactions (ADRs) are potentially avoidable causes of morbidity, mortality, and healthcare utilisation [1]. The Quality in Australian Healthcare study estimated that 51% of ADRs were preventable, 18% of ADRs spent more than 10 extra days in the hospital (range 0–120 days), and 19% of ADRs resulting in permanent disabilities, such as kidney, liver, neurological, and cardiovascular complications [2]. In the UK, the median length of stay (LOS) for patient episodes with ADRs was 20 days (IQR 12–35 days), significantly longer than the median LOS of 8 days (IQR 5–13 days) for episodes without ADRs (*p* < 0.01). ADRs directly increased LOS in 27.0% of episodes, accounting for 4.0% of all inpatient episodes and 1.9% of bed days. The median increase in LOS for these episodes was 4 days (2–7 days) [3]. A recent systematic review revealed that, in high-income countries, the average cost per ADR case ranged from USD 2908.7 to USD 12,129.9, and in low-income countries, from USD 65.0 to USD 581.7 [4].

Disease-modifying antirheumatic drugs (DMARDs) play a crucial role in the management of a wide range of autoimmune inflammatory rheumatic Diseases, including but not limited to Rheumatoid Arthritis (RA), Psoriatic Arthritis, Systemic Lupus Erythematosus, Juvenile Idiopathic Arthritis, and Inflammatory Bowel Disease. These drugs have demonstrated good efficacy and their utilisation has increased over time [5]. RA has the highest prevalence, utilisation of DMARDs, and reports of ADRs compared to other rheumatic diseases. A study of 12,626 cases in a rheumatologic outpatient clinic found that the most common rheumatic diseases were RA (47.30%), spondyloarthropathies (17.23%), SLE (8.10%), gout (7.84%), and vasculitis (6.83%) [6].

ADRs are the second leading cause of discontinuing disease-modifying antirheumatic drugs (DMARDs) in RA patients. With up to 27% of RA patients, leading to exacerbated RA symptoms, inflammation, and the potential requirement of expensive medications [7, 8]. Consequently, it is essential to identify, address, and prevent ADRs in order to effectively assist rheumatic patients, especially those suffering from RA [9]. However, real-world data on ADRs in RA patients is scarce, making difficult to identify ADRs, determine risk factors, and tailored preventive strategies [10]. Without understanding ADRs in RA patients, it is a challenge to enhance care, plan treatments, and ensure RA is managed safely [11].

The Therapeutic Goods Administration (TGA) in Australia is responsible for managing the Drug Adverse Event Notifications (DAEN) [12]. The DAEN database was assembled from ADR reports obtained from health professionals, individuals, and manufacturers [12]. It is an excellent source for tracing and assessing ADRs in individuals with autoimmune rheumatic diseases, such as RA, who were prescribed DMARDs. Researchers can employ it to determine the prevalence of ADRs, identify risk factors and any trends.

Similarly, Food and Drug Administration Adverse Event Reporting System (FAERS) in the United States (US) can monitor and take apart the safety aspects of drugs and medical products [13]. The aim of this study is to determine ADR data related to anti-rheumatics drugs, evaluate the consistency of ADR reporting, and detect any discrepancies in the safety profiles of DMARDs between Australia and the US by comparing the data from the Australian DAEN database with the US FAERS.

## Methods

This a retrospective comparative study and we applied the Strengthening the Reporting of Observational Studies in Epidemiology (STROBE) guidelines [14] to ensure the accuracy and completeness of reporting (Online Resource 1). Individual case reports for selected DMARDs were obtained for the DAEN database in Western Australia from 1995 and 2015 (Table 1). This time frame was selected to capture a substantial amount of data and provide a comprehensive analysis of the association between DMARDs and ADRs. By examining a two-decade period, the study aims to identify any long-term patterns or trends in medication safety. The DAEN database includes de-identified case reports that contain information on the sex, age, potential adverse reactions to medications relationship (“suspected”, “concomitant”, and “interacting”), causality, individual case safety report (ICSR) status, case narrative, sender details, year, and state [12]. Each of the 232 separate reports in the DAEN includes multiple ADRs and involves multiple medications classified by the TGA as suspected, concomitant, or interacting.

**Table 1:** Disease-modifying antirheumatic drugs list requested from the therapeutic goods administration for individual case reports of adverse drug reactions in Western Australia (1995–2015)

DMARDs	Drug class	Approved indication by TGA
Conventional DMARDs		
1. Azathioprine	Immunosuppressants	Rheumatoid arthritis, Crohn's disease, Ulcerative colitis, Autoimmune hepatitis, Dermatomyositis
2. Cyclophosphamide	Immunosuppressants	Rheumatoid arthritis, Systemic lupus erythematosus, Vasculitis, Nephrotic syndrome
3. Cyclosporine	Immunosuppressants	Rheumatoid arthritis, Psoriasis, Atopic dermatitis
4. Hydroxychloroquine	Immunosuppressants	Rheumatoid arthritis, Systemic lupus erythematosus, Malaria prophylaxis and treatment
5. Leflunomide	Immunosuppressants	Rheumatoid arthritis
6. Methotrexate	Immunosuppressants	Rheumatoid arthritis, Psoriasis, Crohn's disease, Juvenile idiopathic arthritis
7. Penicillamine	Immunosuppressants	Rheumatoid arthritis, Wilson's disease
8. Sodium aurothiomalate	Immunosuppressants	Rheumatoid arthritis
9. Sulfasalazine	Immunosuppressants	Rheumatoid arthritis, Inflammatory bowel disease
Biological DMARDs		
10. Rituximab	B-cell targeted therapy	Rheumatoid arthritis, non-Hodgkin lymphoma
11. Adalimumab	Tumor Necrosis Factor inhibitors	Rheumatoid arthritis, Ankylosing spondylitis, Psoriatic arthritis, Crohn's disease, Ulcerative colitis, Hidradenitis suppurativa, Plaque psoriasis
12. Certolizumab	Tumor Necrosis Factor inhibitors	Rheumatoid arthritis, Psoriatic arthritis, Ankylosing spondylitis, Crohn's disease, Plaque psoriasis
13. Etanercept	Tumor Necrosis Factor inhibitors	Rheumatoid arthritis, Juvenile idiopathic arthritis, Psoriatic arthritis, Ankylosing spondylitis, Plaque psoriasis
14. Golimumab	Tumor Necrosis Factor inhibitors	Rheumatoid arthritis, Ankylosing spondylitis, Psoriatic arthritis, Ulcerative colitis, Crohn's disease
15. Infliximab	Tumor Necrosis Factor inhibitors	Rheumatoid arthritis, Ankylosing spondylitis, Psoriatic arthritis, Crohn's disease, Ulcerative colitis, Psoriasis
16. Tocilizumab	Interleukin-6 inhibitors	Rheumatoid arthritis, Juvenile idiopathic arthritis, Giant cell arteritis

*DMARDs* disease-modifying antirheumatic drugs, *TGA* Therapeutic goods administration

Also, we compared DAEN data with the US FAERS to measure the disproportionality using the Proportional Reporting Ratio (PRR) for each DMARD reported ADRs versus all other drugs registered [15]. The data in FAERS are divided into seven groups, which include patient demographic and administrative information, drug information, preferred terms for the events (PTs), patient outcomes for the event, indications of use for the reported drugs, therapy start dates and end dates, and report sources for the event [16].

### Participants

The study included 232 patients from Western Australia who had used TGA approved DMARDs for treatment of immune-mediated conditions. The study was sufficiently powered to detect statistically significant associations between specific DMARDs and ADRs.

### Statistical methods

Non-parametric variables were reported as median values with an interquartile range. Continuous variables are analysed using the Kruskal–Wallis test (for non-normally distributed data). While categorical variables were compared using the chi-square test for independence. In the study's disproportionality analysis, the Medical Dictionary for Regulatory Activities (MedDRA) was used to classify cases of ADRs linked to the use of DMARDs in the Western Australian DAEN databases and US FAERS.

The Proportional Reporting Ratios (PRR) was employed to investigate reporting associations between DMARDs and ADRs [15]. It is calculated by dividing the proportion of reports for a specific drug-event combination by the proportion of reports for all other drug–event combinations [16]. An association was considered statistically significant when the lower limit of the 95% CI was above 1.0, indicating a higher-than-expected reporting of the ADR for the specific drug.

The ADRs considered clinically significant according to the criteria of Evans (*n* > 2, Chi-square > 4, PRR > 2) [15] are referred to as "True ADRs". Spearman's correlation coefficient was used to examine the association between US FAERS pharmacovigilance data PRRs and Western Australia DAEN based on the Shapiro–Wilk test results. The assumption of normality was assessed with the Shapiro–Wilk test for both datasets. The Mann–Whitney *U* test was used to compare statistically between US FAERS pharmacovigilance data PRRs and Western Australia DAEN as well as the distribution of ADRs count between conventional and biological DMARDs in Western Australia DAEN. All statistical analyses were performed in R version 4.3.0, with statistical significance set at *P*-value < 0.05.

### Ethics

The data used in this study were obtained from the Australian TGA and US FDA public domains, which are administered by the Australian Government Department of Health and the US Department of Health and Human Services, respectively. The data are de-identified, publicly available and did not identify any patient, so ethics approval was not required.

## Results

Reports in DAEN involved 58.2% females with median age of 61 years old (IQR: 49, 69) (Table 2). The majority of reported ADRs were possibly causally related to DMARD use, with 82.7% of cases classified as serious ICSR. Serious adverse events increased with age, primarily in ≤ 19 years, those aged 70–79, and individuals over 85. However, these age groups had the lowest rates of ADRs reported. Health professionals were the primary reporting source of ADRs (82.7%).

**Table 2:** Patient characteristics of Individual Case Safety Reports for DMARDs usage in Western Australia (1995–2015)

Characteristics	*N* = 232	Female, *N* = 135 (58.2%)	Male, *N* = 90 (38.8%)	Not Specified, *N* = 7 (3.0%)	*p*-value
Age [Median (IQR)]	61 (49, 69)	62 (47, 70)	59 (50, 65)	67 (33, 69)	0.819
Age groups					< 0.001
≤ 19	21 (9.1%)	13 (9.6%)	6 (6.7%)	2 (28.5%)	
20–29	6 (2.6%)	5 (3.7%)	1 (1.1%)	0 (0%)	
30–44	25 (10.8%)	15 (11.1%)	10 (11.1%)	0 (0%)	
45–59	59 (25.4%)	28 (20.7%)	31 (34.5%)	0 (0%)	
60–69	73 (31.4%)	41 (30.4%)	28 (31.1%)	4 (57.1%)	
70–84	42 (18.1%)	29 (21.5%)	12 (13.3%)	1 (14.3%)	
85 +	6 (2.6%)	4 (3%)	2 (2.2%)	0 (0%)	
RA patients	22 (9.5%)	17 (13%)	5 (5.6%)	0 (0%)	0.146
Causality of ADRs					0.760
Causality certain	15 (6.5%)	9 (6.7%)	6 (6.7%)	0 (0%)	
Causality possible	192(82.7%)	109 (80.7%)	77 (85.6%)	6 (85.7%)	
Causality probable/likely	25 (10.8%)	17 (12.6%)	7 (7.7%)	1 (14.3%)	
Serious ICSR	81 (34.9%)	44 (32.6%)	35 (38.9%)	2 (28.6%)	0.586
Sender					0.653
Health Professionals	191 (82.7%)	106 (79.3%)	79 (87.8%)	6 (85.7%)	
Pharmaceutical company	34 (14.7%)	23 (17.0%)	10 (11.1%)	1 (14.3%)	
Patients	6 (2.6%)	5 (3.7%)	1 (1.1%)	0 (0%)	

*RA* Rheumatoid Arthritis, *ADRs* Adverse drug reactions, *ICSR* Individual Case Safety Report, *DMARDs* Disease-modifying antirheumatic drugs

In the 232 separate DAEN reports, each ICSR contributes to multiple ADRs and involved multiple medications classified as suspected, concomitant, or interacting. Accordingly, the study analysed the association between 1551 ADRs and medications reported from the selected DMARDs (Online resource 2). Out of these, 637 ADRs (41.1%) were related to DMARDs approved for the treatment of rheumatic autoimmune diseases. Conventional and biologic DMARDs responsible for 25.9% and 15.2% of the ADRs involving 402 and 235 diverse types of ADRs (Online resource 3).

### ADR incidence for conventional and biologic DMARDs

The highest incidence of ADRs DAEN was for four conventional DMARDs methotrexate, leflunomide, azathioprine, Sulfasalazine (Table 3). The ADRs most associated with methotrexate were attention disturbance, dyspnea, and headache, which a higher incidence in females (78.6%) than males (19.8%) (Online resource 4). Among biologics, Infliximab had the highest number of ADRs reported. The most frequently ADRs linked to Infliximab were flashing and nausea, both more commonly observed in older females (aged 60–69) and younger males (≤ 19) over the study period (Online resource 5).

**Table 3:** Incidence of adverse drug reactions from the usage of conventional and biological DMARDs based on DAEN database in Western Australia state (1995–2015)

DMARDs	ADRs frequency	Percentage of total ADRs	Suspected for ADRs	Concomitant for ADRs	Interacting for ADRs
1- Methotrexate	126	8.1	108	18	0
2- Leflunomide	105	6.8	96	9	0
3- Azathioprine	75	4.8	74	0	1
4- Sulfasalazine	58	3.7	55	3	0
5- Infliximab	54	3.5	52	2	0
6- Abatacept	49	3.2	49	0	0
7- Etanercept	44	2.8	41	3	0
8- Tocilizumab	33	2.1	33	0	0
9- Rituximab	32	2.1	32	0	0
10- Adalimumab	22	1.4	21	1	0
11- Hydroxychloroquine	19	1.2	14	5	0
12- Cyclophosphamide	15	1.0	15	0	0
13- Cyclosporine	4	0.3	3	1	0
14- Golimumab	1	0.1	1	0	0
15- Certolizumab	0	0	0	0	0
16- Penicillamine	0	0	0	0	0

*ADRs* Adverse drug reactions, *DMARDs* Disease-modifying antirheumatic drugs

**Fig. 1:**
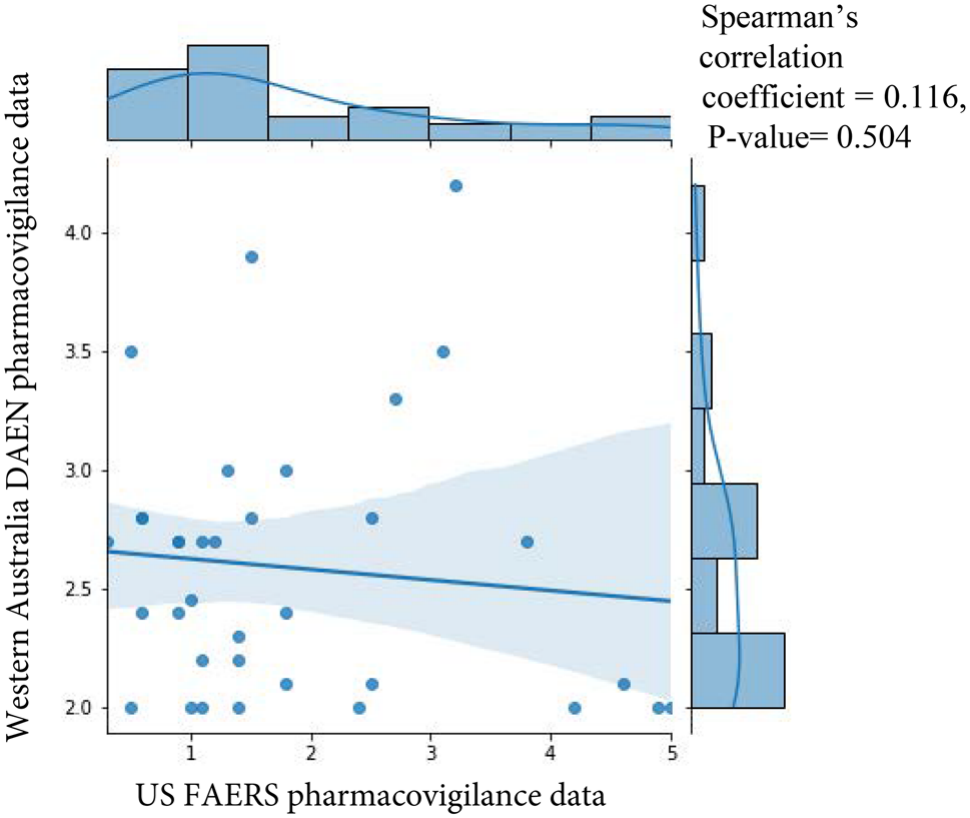
Scatter plot for the correlation between US FAERS pharmacovigilance data and Western Australia DAEN notifications in the study. US FAERS = United State Food and Drug Administration Adverse Event Reporting System

The top ten reported ADRs in DAEN were pyrexia, diarrhea, paraesthesia, and hepatic function abnormal levels, as compared to the US FAERS pharmacovigilance data during the same period (Table 4). The FAERS and the DAEN datasets were assessed for normality using the Shapiro–Wilk test, which returned p-values less than 0.05, indicating a non-normal distribution. Subsequently, Spearman's correlation analysis was conducted as appropriate method. PRR values from the FAERS data and the DAEN data (Fig. 1) showed a weak and negative relationship (Spearman's correlation coefficient =  − 0.116, p = 0.504), suggesting that the PRR values in the two datasets differ. Each point in a scatter plot represents a combination of DMARD and ADR. The diagram confirms the weak negative association, but the scattered dots indicate its weakness.

**Table 4:** The ten most frequently reported true ADRs for with the usage of DMARDs on DAEN database in Western Australia state compared with US FAERS pharmacovigilance data (1995–2015)

DMARDs	ADRs (PTs)	MedDRA SOC	Western Australia DAEN notifications [PRR (95% CI)]	US FAERS pharmacovigilance data (PRR) [PRR (95% CI)]
Azathioprine	Pyrexia	General disorders	4.2 (3.5–5)	3.2 (3–3.4)
Leflunomide	Diarrhea	Gastrointestinal disorders	3.9 (3.1–4.6)	1.5 (1.3–1.6)
Sulfasalazine	Pyrexia	General disorders	3.5 (2.6–4.4)	3.1 (2.8–3.5)
Abatacept	Paraesthesia	Nervous system disorders	3.5 (2.6–4.5)	0.5 (0.2–1.6)
Leflunomide	Hepatic function abnormal	Hepatobiliary disorders	3.3 (2.5–4.1)	2.7 (2–3.7)
Azathioprine	Chills	General disorders	3 (2.1–3.9)	1.8 (1.5–2.2)
Azathioprine	Vomiting	Gastrointestinal disorders	3 (2.1–3.9)	1.3 (1.1–1.4)
Leflunomide	Nausea	Gastrointestinal disorders	2.8 (1.9–3.7)	2.5 (2.4–2.6)
Sulfasalazine	Pruritus	Skin and subcutaneous tissue disorders	2.8 (1.8–3.8)	1.5 (1.3–1.7)
Abatacept	Dizziness	Nervous system disorders	2.8 (1.8–3.8)	0.6 (0.5–0.7)

*DMARDs* Disease-Modifying Antirheumatic Drugs, *ADRs* Adverse drug reactions, *DAEN* Drug Adverse Event Notifications, *US FAERS* United State Food and Drug Administration Adverse Event Reporting System, *PT* Preferred Term, *MedDRA* Medical Dictionary for Regulatory Activities Terminology, *SOC* System Organ Class, *PRR* Proportional Reporting Ratio, 95% *CI* 95% Confidence Intervals

### The association analysis

**Fig. 2:**
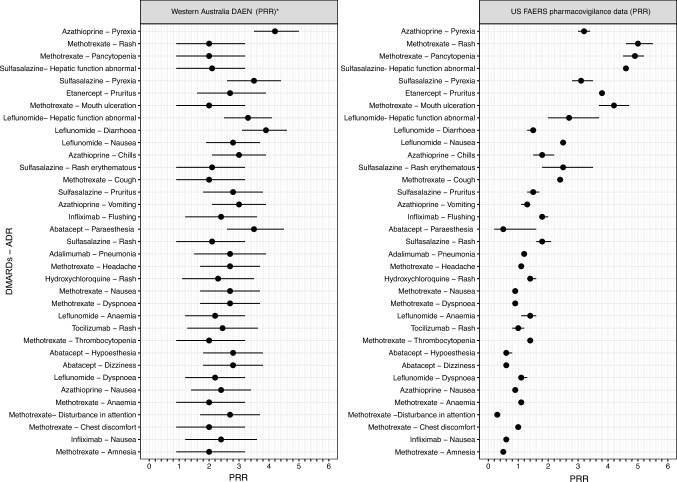
A comparative analysis of clinically significant adverse drug reactions of disease-modifying antirheumatic drugs in Western Australian DAEN compared with US FAERS pharmacovigilance data between 1995 and 2015. *According to the criteria of Evans 2001 to be likely ADR (*n* > 2, Chi-square > 4, PRR > 2) [15], ADR = Adverse drug reaction, DMARDs = disease-modifying antirheumatic drugs, PRR = Proportional Reporting Ratio, 95% CI = 95% Confidence Interval, US FAERS = United State Food and Drug Administration Adverse Event Reporting System

DAEN and FAERS ADR associations are illustrated in Fig. 2. Azathioprine is strongly associated with “pyrexia” in DAEN, with a PRR of 4.2 across the study period (Fig. 2). While the lowest PRR was “vomiting” related with leflunomide (1.1). The highest PRR value in FAERS was for “Rash” related with methotrexate (5.0) over the same study period. In contrast, the lowest PRR in FAERS was for “disturbance in attention” associated with methotrexate use, with a PRR of 0.3.

FAERS data demonstrate a substantial relationship with rash, pancytopenia, and mouth ulcers with methotrexate utilisation, whereas DAEN data suggest disruption in attention, dyspnea, and headache associated with methotrexate usage. Leflunomide had the greatest PRR (3.3) in the DAEN for hepatic function abnormalities, whereas sulfasalazine had the highest PRR for hepatic function abnormalities (4.6) in the FAERS. Based on matching US FAERS data, etanercept was most associated with pruritus (PRR = 3.8), while abatacept was strongly associated with paraesthesia (PRR = 3.8) in DAEN. There were no statistically significant differences in the PRR estimates derived from DAEN and FAERS (*p* = 0.15).

### Trend analysis of ADRs associated with DMARDs overtime

**Fig. 3:**
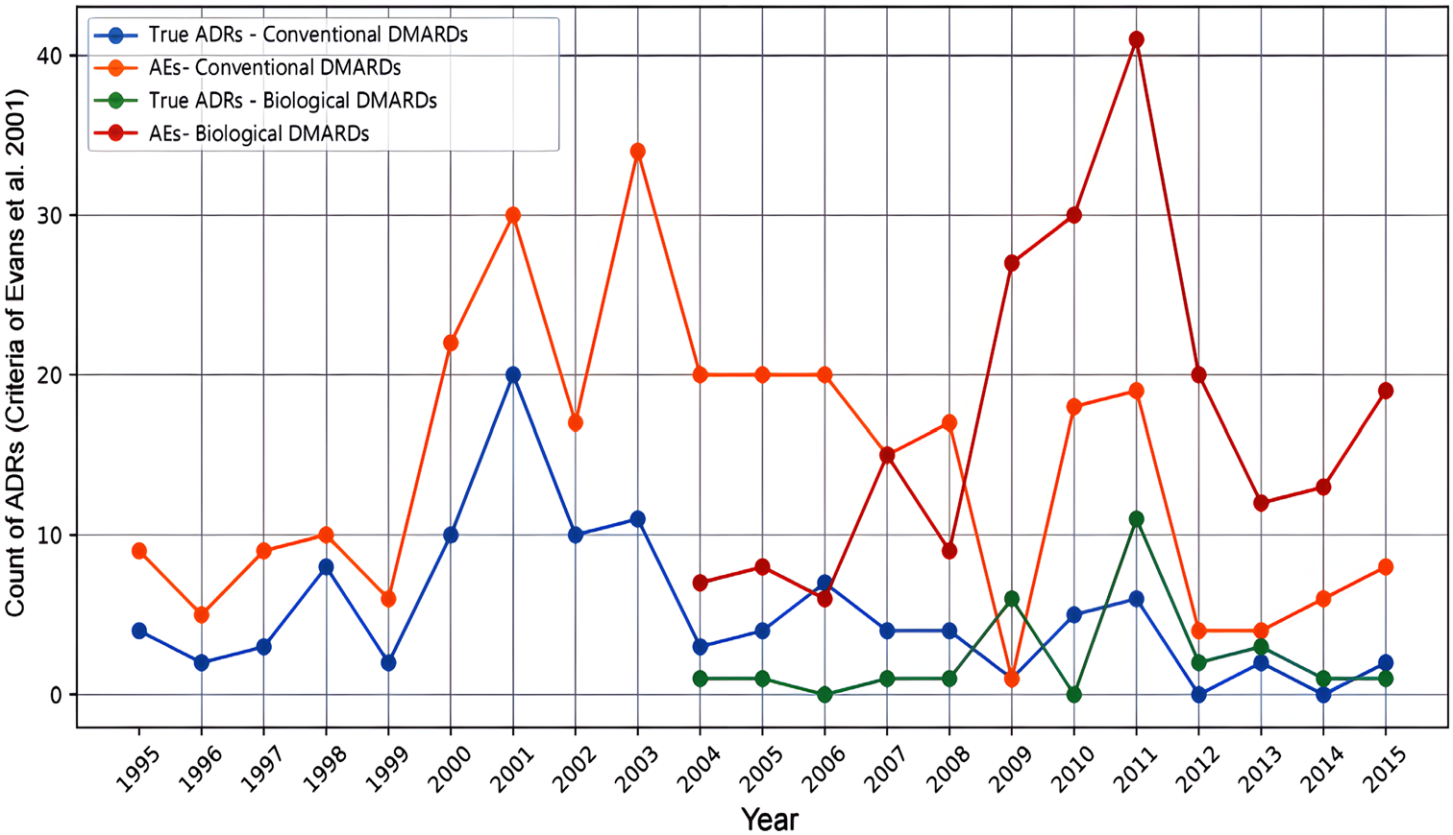
The trend analysis of ADRs in the DAEN database related to type DMARDs utilisation in Western Australia (1995–2015). *According to the criteria of Evans 2001 to be likely ADR (*n* > 2, Chi-square > 4, PRR > 2) [15], ADRs = Adverse drug reactions, AEs = Adverse events, DMARDs = disease-modifying antirheumatic drugs

The trend analysis of ADRs in DAEN is presented in (Fig. 3) showed no statistically significant difference in incidence of ADRs between conventional and biological DMARDs over time. Also, it shows different trendlines depicting the frequency of ADR occurrences for combinations of DMARDs over time. The graph clearly illustrates trends within each category and whether there are true ADRs or adverse events (AEs). It demonstrates the variation in total ADR counts for DMARD groups over time. For biological DMARDs, a clear trend was not observed until 2004. In 2001 and 2011, there was an increase in true ADRs for conventional and biological DMARDs, respectively. These spikes can be attributed to reported specific true ADRs with certain DMARDs. In 2001, there was an increased incidence of disturbance in attention and anemia reported with methotrexate and leflunomide use (Online resource 6). Similarly in 2011, paraesthesia and hypoesthesia were reported for Abatacept and methotrexate use (Online resource 7). In contrast, there was a decline in ADRs incidence for conventional and biological DMARDs in 2013.

### Relationship between true ADRs and type of DMARDs

We examined the relationship between true ADRs and type of DMARDs by Chi-Squared test that resulting a significant association (p < 0.01) between the two variables (Online resource 8). The Odds Ratio (OR) is 2.71, with a 95% CI 1.72 and 4.26, showing that patients treated with biological DMARDs have about 2.7 times higher odds of reporting true ADRs compared to those treated with conventional DMARDs (Online resource 8).

## Discussion

This first study examines the ADRs to DMARDs in Western Australia from 1995 to 2015. The study found 232 reports in the DAEN, involving women aged 45–69. Methotrexate, leflunomide, and azathioprine had the highest number of ADRs reported, mostly related to gastrointestinal issues. Patients treated with biological DMARDs had approximately 2.7 times higher odds of reporting true ADRs in DAEN compared to those treated with conventional DMARDs. The study also confirms consistency between the Western Australian DAEN and US FAERS data.

Over 20 years, the recorded number of ADRs related to DMARDs indicates a generally well-tolerated and manageable toxicity profile, although evidence of under-reporting [17]. In Australia, a retrospective analysis examined 1547 calls with an Australian medical call center involving both conventional and biologic DMARDs from September 2002 to June 2010 [18]. The study revealed that most patients' enquiries and concerns regarding DMARDs focused on their effectiveness and appropriate dosage rather than safety considerations [18]. Some studies suggest that DMARDs rarely cause ADRs [11, 19, 20]. Also, most AEs are minor and usually do not require stopping the treatment [19]. Positive experiences with DMARDs show that doctors can address patient concerns regarding potential harm and customise therapy based on patients' beliefs, lifestyles, and goals [20].

More than half of the DMARD patients in the ICSR were female (58.2%), raising questions about potential gender differences in ADR-related DMARD usage for RA patients, especially changes in treatment regimens indicating ADRs [21]. For instance, a study found that male RA patients responded better to methotrexate than females [22]. In our study, females reported a higher number of ADRs for methotrexate and infliximab compared to males. This suggests that females report more ADRs and report them more often to healthcare professionals [23].

Hormone levels and body functions in men and women may be responsible for the different reactions to DMARDs. [10, 24, 25]. A study has found that 72 females and 28 males out of 100 RA patients endured more severe ADRs [10]. Further confirmation of this came from studies conducted in 2023, which observed that female RA patients had a reduced response to DMARDs, implying that women may be more prone to ADRs [24].

It has been studied that around half of the ADRs require modifications to primary care treatment [21]. In the early treatment of RA, these ADRs altered treatment options, making distinctive treatments for men and women [24]. The MARI study showed that women were especially cautious when considering DMARDs [25]. Such findings help to distinguish gender-related risks from treatments.

Yet, the current DMARD doses do not take into consideration the potential for disparate responses between men and women to these medications [26]. EULAR has studied the safety of DMARDs over the years [27]; however, there is no research on the effect of gender on RA treatments [26]. In our study, approximately 83% of medical personnel recorded ADRs, demonstrating the importance of their part in monitoring and submitting these events. Nonetheless, the outlook of healthcare personnel in Australia and the U.S. may hinder ADRs reporting [28–30].

Methotrexate, a common DMARD, has been shown to cause liver damage, bone marrow suppression, and gastrointestinal disorders [31]. Similarly, infliximab may possibly cause serious infections and reactions [32]. Our study into the toxicity of DMARDs ascertained a high prevalence of ADRs. Nevertheless, these drugs are often prescribed to those with severe autoimmune illnesses like RA, as the benefits usually surpass the hazards. The characteristics of patients, multiple medication uses, and drug responses can all modify how DMARDs respond adversely [33, 34]. To avoid these risks, practitioners must monitor their patients and adjust their treatment [35].

This highlights the importance of healthcare professionals being aware of and actively managing the ADRs of DMARDs. This includes comprehensive assessment [36], safe prescribing [37], risk communications [36], monitoring [35], education [36], post-marketing surveillance [38], and reporting ADRs to the relevant authorities.

The PRR values in both the DAEN and FARES databases revealed no major discrepancies, indicating consistent detection of ADRs. Azathioprine had the highest number of true ADRs, based on Evans’ criteria and previous studies [11, 39]. A Pan American Journal of Public Health study reported a higher incidence of ADRs with Azathioprine than other DMARDs [11]. Similarly, a Cochrane Database System Review found more ADRs with azathioprine compared to other DMARDs [39].

Several elements can be associated with specific ADRs to DMARDs, including gene expression regulation, clinical characteristics, non-adherence, comorbidities, and patient differences in epigenetics [34, 40]. Some drugs have properties that could increase the chance of having ADRs [41]. Understanding these areas could help practitioners predict ADRs and enhance therapy choices. More research is needed to understand the association between certain ADRs and DMARDs. Applying these signs could facilitate the formation of tools or models to predict who could be at elevated risk [41]. In our study, patients using biological DMARDs reported true ADRs 2.7 times more than those on conventional DMARDs. This aligns with a retrospective cohort study's conclusion that biological DMARDs have more ADRs than non-biological DMARDs [11]. Moreover, certain biologic DMARDs, such as tocilizumab, rituximab, and infliximab, displayed a greater association with ADRs [11]. It is critical to determine if biological DMARDs cause severe gastrointestinal disturbances and frequent hospital ADRs. This data can be of aid when making decisions about treatment and tracking patients taking conventional and biological DMARDs. Obtaining further data from Western Australian hospitals might expose the range and intensity of DMARD-related ADRs that could have an impact on treatment decisions, patient monitoring, and outcomes.

### Strengths

The study emphasised the importance of pharmacovigilance in Australia and the USA for monitoring medication safety when using DMARDs for RA treatment. The Australian DAEN and US FAERS databases provided comprehensive patient data on ADRs, ensuring a diverse representation of patients, and improving the study’s generalisability. Using these data sources, the study avoided potential biases associated with participant recruitment and selection in clinical trials.

## Limitations

The DAEN from Australia and the FAERS from the US have several limitations. Such data may be influenced by individuals voluntary reporting. Furthermore, severe or rare ADRs are more likely to be overestimated. Minor or less severe AEs may not be reported sufficiently. Keeping track of the quality and completeness of the data can be challenging. Also, discrepancies in the accuracy and uniformity of reports reduce their reliability. RA medications endorsed in Western Australia may not be compatible with other populations or locations. To validate these findings, larger and more varied groups of patients need to be studied. Medical personnel typically detect these AEs more often. Hence, patients may be less likely to report incidents. Possible side effects could be identified with the reporting platform of Western Australia's DAEN. Additionally, reporting to the Western Australian DAEN may not capture all ADRs. Therefore, it's essential to interpret the findings cautiously and consider other data sources for validation. The reliability of PRR estimates is influenced by factors, such as database size, ADR report quality, and the prevalence of ADRs and DMARDs in the population. Therefore, PRR should be used alongside other evidence and clinical judgment. The decrease in adverse ADRs on conventional and biological DMARDs in 2013 remains unexplained. We observed a corresponding decline in DMARDs usage in our previous study in Western Australia [42] and nationally [43].

## Conclusion

Methotrexate and infliximab are commonly reported ADRs in female than males in Western Australia, but less severe compared with other conventional DMARDs such as Azathioprine. Although there was no significant difference in incidence of ADRs between conventional and biological DMARD treatment over study period, patients treated with biological DMARDs are 2.7 times likely to report the true ADRs compared to those treated with conventional DMARDs. To ensure the validity and generalisability of our findings, it is crucial to conduct further research using larger sample sizes and linked data from more diverse populations.

### Supplementary Information

Below is the link to the electronic supplementary material.

Supplementary file1 (PDF 288 KB)

## Data Availability

In line with the principles of open data sharing, the data used in this study may be made available for further research upon reasonable request to the corresponding author.
