# Supplementary material for: The temporal association between adverse drug reactions and antirheumatic drugs utilisation in Western Australia: a retrospective study from real-world data (1995–2015)
Source: Rheumatol Int. 2024 Apr 14;44(6):1089–99. doi: 10.1007/s00296-024-05588-3 (PMC11108947; doi:10.1007/s00296-024-05588-3)
Supplement: Supplementary file 1 — Supplementary file1 (PDF 288 KB) [file 296_2024_5588_MOESM1_ESM.pdf]

The Temporal Association Between Adverse Drug Reactions and Antirheumatic Drugs Utilisation in Western Australia: A Retrospective Study from Real-World Data (1995–2015)

Khalid B Almutairi[1,2*] · Charles A Inderjeeth[1,3] · David B Preen[4] · Helen I Keen[1,5] · Johannes C Nossent[1,3]

[1] School of Medicine, The University of Western Australia, Perth, Western Australia, Australia

[2] Pharmacy Department, King Fahd Specialist Hospital, Burydah, Al Qassim, Saudi Arabia

[3] Rheumatology Department, Sir Charles Gairdner and Osborne Park Health Care Group, Perth, Western Australia, Australia

[4] School of Population and Global Health, University of Western Australia, Perth, Western Australia, Australia

[5] Rheumatology Department, Fiona Stanley Hospital, Murdoch, Western Australia, Australia

* First and corresponding author: Dr Khalid B Almutairi

* Corresponding author

Khalid B Almutairi

E-mail: khalid.almutairi@uwa.edu.au

Drug Safety Journal

Supplementary online materials

Online Resources 1

STROBE Statement—checklist of items that should be included in reports of observational studies

	Item No	Recommendation	Page No
Title and abstract	1	(*a*) Indicate the study's design with a commonly used term in the title or the abstract	1
		(*b*) Provide in the abstract an informative and balanced summary of what was done and what was found	3
Introduction			
Background/rationale	2	Explain the scientific background and rationale for the investigation being reported	4-5
Objectives	3	State specific objectives, including any prespecified hypotheses	5
Methods			
Study design	4	Present key elements of study design early in the paper	5-6
Setting	5	Describe the setting, locations, and relevant dates, including periods of recruitment, exposure, follow-up, and data collection	5-6
Participants	6	(*a*) *Cohort study*—Give the eligibility criteria, and the sources and methods of selection of participants. Describe methods of follow-up<br>*Case-control study*—Give the eligibility criteria, and the sources and methods of case ascertainment and control selection. Give the rationale for the choice of cases and controls<br>*Cross-sectional study*—Give the eligibility criteria, and the sources and methods of selection of participants	6-7
		(*b*) *Cohort study*—For matched studies, give matching criteria and number of exposed and unexposed<br>*Case-control study*—For matched studies, give matching criteria and the number of controls per case	6-7
Variables	7	Clearly define all outcomes, exposures, predictors, potential confounders, and effect modifiers. Give diagnostic criteria, if applicable	6-7
Data sources/ measurement	8*	For each variable of interest, give sources of data and details of methods of assessment (measurement).	*5-6*

		Describe comparability of assessment methods if there is more than one group	
Bias	9	Describe any efforts to address potential sources of bias	14
Study size	10	Explain how the study size was arrived at	5
Quantitative variables	11	Explain how quantitative variables were handled in the analyses. If applicable, describe which groupings were chosen and why	6-7
Statistical methods	12	(*a*) Describe all statistical methods, including those used to control for confounding	6-7
		(*b*) Describe any methods used to examine subgroups and interactions	6-7
		(*c*) Explain how missing data were addressed	NA
		(*d*) *Cohort study*—If applicable, explain how loss to follow-up was addressed<br>*Case-control study*—If applicable, explain how matching of cases and controls was addressed<br>*Cross-sectional study*—If applicable, describe analytical methods taking account of sampling strategy	NA
		(*e*) Describe any sensitivity analyses	NA

Results

Participants	13*	(a) Report numbers of individuals at each stage of study—eg numbers potentially eligible, examined for eligibility, confirmed eligible, included in the study, completing follow-up, and analysed	Table 2
		(b) Give reasons for non-participation at each stage	NA
		(c) Consider use of a flow diagram	NA
Descriptive data	14*	(a) Give characteristics of study participants (eg demographic, clinical, social) and information on exposures and potential confounders	Table 2
		(b) Indicate number of participants with missing data for each variable of interest	NA
		(c) *Cohort study*—Summarise follow-up time (eg, average and total amount)	Figure 3
Outcome data	15*	*Cohort study*—Report numbers of outcome events or summary measures over time	*Table 1 & Figure 3*
		Case-control study—Report numbers in each exposure category, or summary measures of exposure	*NA*
		Cross-sectional study—Report numbers of outcome events or summary measures	*NA*

Main results	16	(*a*) Give unadjusted estimates and, if applicable, confounder-adjusted estimates and their precision (eg, 95% confidence interval). Make clear which confounders were adjusted for and why they were included	Table 4
		(*b*) Report category boundaries when continuous variables were categorized	Table 2 & 3
		(*c*) If relevant, consider translating estimates of relative risk into absolute risk for a meaningful time period	Table 3, online resource 3
Other analyses	17	Report other analyses done—eg analyses of subgroups and interactions, and sensitivity analyses	Table 3, online resource 3
Discussion			
Key results	18	Summarise key results with reference to study objectives	11-13
Limitations	19	Discuss limitations of the study, taking into account sources of potential bias or imprecision. Discuss both direction and magnitude of any potential bias	14-15
Interpretation	20	Give a cautious overall interpretation of results considering objectives, limitations, multiplicity of analyses, results from similar studies, and other relevant evidence	14-15
Generalisability	21	Discuss the generalisability (external validity) of the study results	15
Other information			
Funding	22	Give the source of funding and the role of the funders for the present study and, if applicable, for the original study on which the present article is based	2

Reference:
von Elm E, Altman DG, Egger M, Pocock SJ, Gøtzsche PC, Vandenbroucke JP (2007) The Strengthening the Reporting of Observational Studies in Epidemiology (STROBE) statement: guidelines for reporting observational studies. Lancet 370(9596):1453-1457. https://doi.org/10.1016/s0140-6736(07)61602-x

Online resource 2:

ADRs

		Frequency	Percent	Valid Percent	Cumulative Percent
Valid	Non DMARDs	914	58.9	58.9	58.9
	DMARDs	637	41.1	41.1	100.0
	Total	1551	100.0	100.0	

Online resource 3:

ADRs

		Frequency	Percent	Valid Percent	Cumulative Percent
Valid	Non DMARDs	914	58.9	58.9	58.9
	Conventional DMARDs	402	25.9	25.9	84.8
	Biological DMARDs	235	15.2	15.2	100.0
	Total	1551	100.0	100.0	

Online resource 4:

Age and Sex and methotrexate ADRs

Sex			Frequency	Percent	Valid Percent	Cumulative Percent
Female	Valid	<= 19	6	6.1	6.1	6.1
		20 - 29	2	2.0	2.0	8.1
		30 - 44	15	15.2	15.2	23.2
		45 - 59	32	32.3	32.3	55.6
		60 - 69	20	20.2	20.2	75.8
		70 - 84	17	17.2	17.2	92.9
		85+	7	7.1	7.1	100.0
		Total	99	100.0	100.0	
Male	Valid	<= 19	1	4.0	4.0	4.0
		30 - 44	4	16.0	16.0	20.0
		45 - 59	12	48.0	48.0	68.0
		60 - 69	4	16.0	16.0	84.0
		70 - 84	4	16.0	16.0	100.0
		Total	25	100.0	100.0	
Not Specified	Valid	70 - 84	2	100.0	100.0	100.0

Online resource 5:

Age and Sex and Infliximab ADRs

Sex			Frequency	Percent	Valid Percent	Cumulative Percent
Female	Valid	<= 19	8	29.6	29.6	29.6
		60 - 69	15	55.6	55.6	85.2
		70 - 84	4	14.8	14.8	100.0
		Total	27	100.0	100.0	
Male	Valid	<= 19	10	40.0	40.0	40.0
		20 - 29	2	8.0	8.0	48.0
		30 - 44	6	24.0	24.0	72.0
		45 - 59	5	20.0	20.0	92.0
		60 - 69	1	4.0	4.0	96.0
		70 - 84	1	4.0	4.0	100.0
		Total	25	100.0	100.0	
Not Specified	Valid	60 - 69	2	100.0	100.0	100.0

Online resource 6:

Conventional DMARDs True ADRs in 2001

		Frequency	Percent	Valid Percent	Cumulative Percent
Valid	Hydroxychloroquine	1	5.0	5.0	5.0
	Leflunomide	7	35.0	35.0	40.0
	Methotrexate	9	45.0	45.0	85.0
	Sulfasalazine	3	15.0	15.0	100.0
	Total	20	100.0	100.0	

Conventional DMARDs True ADRs in 2001

		Hydroxychloroquine Count	Leflunomide Count	Methotrexate Count	Sulfasalazine Count
ADRs	Amnesia	0	0	2	0
	Anaemia	0	3	1	0
	Diarrhoea	0	1	0	0
	Disturbance in attention	0	0	2	0
	Headache	0	0	1	0
	Nausea	0	3	1	0
	Pancytopenia	0	0	1	0
	Pyrexia	0	0	0	2
	Rash	1	0	0	1
	Thrombocytopenia	0	0	1	0

Online resource 7:

True ADRs for DMARD only 2011

		Frequency	Percent	Valid Percent	Cumulative Percent
Valid	Abatacept	9	52.9	52.9	52.9
	Methotrexate	4	23.5	23.5	76.5
	Sulfasalazine	2	11.8	11.8	88.2
	Tocilizumab	2	11.8	11.8	100.0
	Total	17	100.0	100.0	

ADRs

		Frequency	Percent	Valid Percent	Cumulative Percent
Valid	Total	17	100.0	100.0	
	Paraesthesia	4	23.5	23.5	76.5
	Hypoaesthesia	3	17.6	17.6	47.1
	Rash	3	17.6	17.6	94.1
	Dizziness	2	11.8	11.8	17.6
	Rash erythematous	1	5.9	5.9	100.0
	Mouth ulceration	1	5.9	5.9	52.9
	Hepatic function abnormal	1	5.9	5.9	29.4
	Chest discomfort	1	5.9	5.9	5.9
	Headache	1	5.9	5.9	23.5

Online resource 8:

Contingency Tables

Contingency Tables

	DMARDS		
True_ADR	Biological DMARDs	Conventional DMARDs	Total
1 (No)	207	294	501
2 (Yes)	28	108	136
Total	235	402	637

Chi-Squared Tests

	Value	df	p	VS-MPR*
X^2	19.742	1	< .001	3567.274
X^2 continuity correction	18.861	1	< .001	2342.417
Likelihood ratio	21.125	1	< .001	6918.457
N	637			

* Vovk-Sellke Maximum *p* -Ratio: Based the *p* -value, the maximum possible odds in favor of H_1 over H_0 equals 1/(-e *p* log(*p*)) for *p* ≤ .37 (Sellke, Bayarri, & Berger, 2001).

Odds Ratio

		95% Confidence Intervals		
	Odds Ratio	Lower	Upper	p
Odds ratio	2.716	1.728	4.268	
Fisher's exact test	2.712	1.823	∞	< .001

Note. For all tests, the alternative hypothesis specifies that group *1* is greater than *2*.

Nominal

	Value
Contingency coefficient	0.173
Phi-coefficient	0.176
Cramer's V	0.176
Lambda (rows)	0.000
Lambda (columns)	0.000
Lambda (symmetric)	0.000

Ordinal Gamma

		95% Confidence Intervals	
**Gamma**	**Standard Error**	**Lower**	**Upper**
0.462	0.091	0.284	0.640

Kendall's Tau

Kendall's Tau-b	Z	p	VS-MPR*
0.176	4.440	< .001	3514.790
